# The Impact of Bacteriospermia on Semen Parameters: A Meta-Analysis 

**Published:** 2018-06

**Authors:** Vasilios Pergialiotis, Nikoleta Karampetsou, Despina N. Perrea, Panagiotis Konstantopoulos, Georgios Daskalakis

**Affiliations:** 1 Laboratory of Experimental Surgery and Surgical Research N.S. Christeas, National and Kapodistrian University of Athens, Athens, Greece; 2 First Department of Obstetrics and Gynecology, Alexandra Hospital, National and Kapodistrian University of Athens, Athens, Greece

**Keywords:** Sperm, Semen, Infection, Fertility, Bacteriospermia, Meta-Analysis

## Abstract

**Objective:** To evaluate the impact of bacteriospermia on semen parameters.

**Materials and methods:** We used the Medline (1966-2017), Scopus (2004-2017), Clinicaltrials.gov (2008-2017), EMBASE, (1980-2017), LILACS (1985-2017) and Cochrane Central Register of Controlled Trials *CENTRAL *(1999-2017) databases in our primary search along with the reference lists of electronically retrieved full-text papers. Meta-analysis was performed with the RevMan 5.3 software.

**Results:** Eighteen studies were finally included. Men were stratified in two groups, healthy controls (5,797 men) and those suffering from bacteriospermia (3,986 men). Total sperm volume was not affected by the presence of bacteriospermia when all pathogens were analyzed together (MD 0.02 95%CI -0.13,0.17). Both sperm concentration (MD -27.06, 95% CI -36.03, -18.08) and total sperm count (MD -15.12, 95% CI -21.08, -9.16) were significantly affected by bacteriospermia. Decreased rates of normal sperm morphology were also found (MD -5.43%, 95% CI -6.42, -4.44). The percentage of alive sperm was significantly affected by bacteriospermia (MD -4.39 %, 95% CI -8.25, -0.53). Total motility was also affected by bacteriospermia (MD -3.64, 95% CI -6.45, -0.84). In addition to this, progressive motility was significantly affected (MD -12.81, 95% CI -18.09, -7.53). Last but not least, pH was importantly affected (MD 0.03, 95% Cl 0.01, 0.04).

**Conclusion:** Bacteriospermia significantly affects semen parameters and should be taken in mind even when asymptomatic. Further studies should evaluate the impact of antibiotic treatment on semen parameters and provide evidence on fertility outcome.

## Introduction

Bacteriospermia is diagnosed when bacteria in the ejaculate exceed 1000 cfu/ml (1). It is usually the result of acute or chronic bacterial infections and is regarded as a major health care problem which has a negative impact on male fertility (1-3). Specifically, it has been shown that approximately 15% of infertile men have significant number of bacterial pathogens in the sperm (1). Bacterial infections may affect various sites of the male genitourinary system, such as the prostate, the epididymis, the testis and the urethra (1, 3). The most common isolated pathogenic bacteria are Escherichia Coli, Chlamydia trachomatis, Ureaplasmaurealyticum, Mycoplasma, Staphylococci, Streptococci and Enterococcus faecalis (1, 4).On the other hand, the male urinary system is not completely sterile as it has been already shown that certain bacteria, such as Staphylococcus epidermidis, are identified in otherwise healthy reproductive men (4). The impact of different bacteria on sperm quality remains to date unknown (5).

The last decades, the constantly increasing population of infertile couples, has turned scientific interest towards the investigation of the impact of bacteriospermiaon male reproductive ability (6). Various pathophysiologic mechanisms have been investigated to confirm the correlation ofbacteriospermia with seminal parameters, including motility and vitality (7). It is speculated that both the direct bacterial interaction and the participation of the immune competent cells influence spermatogenesis, impair semen function and obstruct the urogenital tract (7, 8). However, the actual impact of each pathogen on seminal parameters remains unknown.

The purpose of our meta-analysis is to accumulate current knowledge in the field and to provide recommendations for clinical practice, as well as new scientific targets for the future.

## Materials and methods

***Study design: ***We used the Preferred Reporting Items for Systematic Reviews and Meta-Analyses (PRISMA) guidelines to design this systematic review (9). Eligibility criteria were predetermined by the authors. Language and date restrictions were avoided during the literature search. All observational studies (both prospective and retrospective, randomized and non-randomized) that reported the impact of bacteriospermia (irrespective of the pathogen) on seminal parameters were held eligible for inclusion and tabulation. Case reports and review articles were excluded. Animal studies were also excluded. 

The study selection took place in three consecutive stages. In the first stage, two researchers (VP, NK) independently reviewed the titles and/or abstracts of all electronic articles to assess their eligibility. Next, the articles that met or were presumed to meet the criteria for inclusion in the present meta-analysis were retrieved in full text. During the third stage, two authors (NK, PK) tabulated the selected indices in structured forms. Potential disagreementsin the evaluation of the methodological quality, retrieval of articles, and statistical analysis were resolved after discussing with the remaining authors.

**Figure 1:**
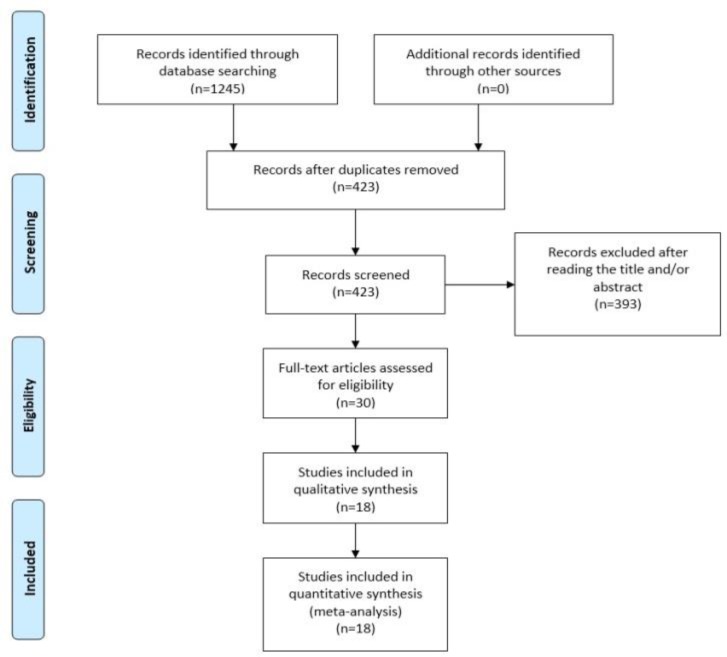
Search plot diagram

***Literature search and data collection:*** We used the Medline (1966-2017), Scopus (2004-2017), Clinicaltrials.gov (2008-2017), EMBASE, (1980-2017), LILACS (1985-2017) and Cochrane Central Register of Controlled Trials *CENTRAL *(1999-2017) databases in our primary search along with the reference lists of electronically retrieved full-text papers. The date of our last search was set at 31^st^December, 2017. Search strategies and results are shown in Figure 1.

Our search strategy included the words semen; sperm; bacteria; bacteriospermia; infection; ureaplasma; mycoplasma; Neisseria; chlamydia; gardnerella; Escherichia coli; streptococcus. The PRISMA flow diagram schematically presents the stages of article selection (Figure 1).

***Quality assessment:*** We assessed the methodological quality of all included studies using the Oxford Level of Evidence criteria and the GRADE list (10). 

***Statistical analysis: ***Statistical meta-analysis was performed with the RevMan 5.3 software (*Copenhagen: The Nordic Cochrane Centre, The Cochrane Collaboration, 2011*). Confidence intervals were set at 95%. We calculated pooled odds ratios (OR), mean differences (MD) and 95% confidence intervals (CI) with the DerSimonian-Laird random effect model due to the significant heterogeneity of included studies (11). Similarly, publication bias was not tested due to the small number of studies and their gross heterogeneity (significant confounders that may influence the methodological integrity of these tests) (12).

***Analyzed indices and subgroup analysis:*** The analyzed indices were tabulated in structured forms, which included significant sperm characteristics, such as sperm volume, Ph, total sperm count, concentration, normal morphology, total motile sperm count, progressive and total motility, sperm vitality and WBC. Subgroup analysis according to the type of pathogen was performed.

## Results

***Excluded studies:*** Twelve studies were excluded from the present meta-analysis as they either did not include a control group of infertile men or did not investigate the outcomes of interest (13-24).

***Included Studies:*** Eighteen studies were finally included (25-42). Men were stratified in two groups, healthy controls (5,797 men) and those suffering from bacteriospermia (3,986 men). In the latter group, 858 patients suffered from mixed bacterial infection, 204 from Chlamydia trachomatis, 640 from Mycoplasma, 2,255 from Ureaplasma Urealyticum, 5 from Ureaplasma Parvum and 24 from Gardnerella vaginalis. The majority of available evidence was drawn from studies of low quality (Level of Evidence 2b and 3b –Table 1).

**Table 1:** Study characteristics

**Date; author**	**Type of study**	**GRADE**	**Inclusion criteria**
1985; Grizard G. et al	Prospective cohort	2b	Men with significant bacteriospermia were included in the study. None of them had any clinical or gormonal abnormalities.
1997; Bussen S.	Prospective cohort	2b	Men who consecutively entered the IVF program of the clinic during a seven-month period in 1995 were included in the study. None of these patients had symptoms of genital tract infection or were treated with antibiotics 4 weeks before the treatment cycle.
2003; L.Knox C.	Prospective cohort	2b	Male partners from couples who participated in an assisted reproductive technology (ART) treatment cycle were included inb the study.
2003; Rodin M.D.	Prospective cohort	2b	Asymptomatic men who were undergoing infertility evaluation were included in the study.
2004; Hosseinzadeh S.	Prospective cohort	2b	Men who attended the University Research Clinic (Jessop Hospital for Women, Sheffield, United Kingdom) for diagnostic semen analysis. All men were undergoing semen analysis as a part of a work-up for infertility suggestions after failing to conceive with their partner after one year of unprotected intercourse.
2005; Sanocka- Maciejewska	Prospective cohort	2b	Men with or without genital tract infection were included in the study. Men had no ability to conceive for at least 2 years of sexual intercourse.
2005; Motrich R.D.	Prospective cohort	2b	Men with Chronic Prostatitis Syndrome, whose age was 20-50 years, were included in the study.
2006; De Barbeyrac B,	Prospective cohort	2b	Men from couples who were undergoing an IVF program between January 1998 and November 2001 were included in the study. Men were between 18 and 55 years old and they didn’t have azoospermia.
2006; Wang Y. et al	Prospective cohort	2b	Men aged 20-45 years who attended the andrology clinic in Shanghai Tonghi Hospital and SanghaiRenji Hospital from March 1, 2001 to March 1, 2003 were included in the study. All men did not present any reproductive abnormalities and had not received any antibiotic treatment.
2008; Gdoura R. et al	Prospective cohort	2b	Men who were attending obstetrics and gynecology clinics in Sfax for infertility were included in the study. All patients did not present any clinical symptoms of genital tract infections except for their infertility health problem.
2009; Andrade- Rocha	Prospective cohort	2b	Men who had history of infertility for at least one year and had never received antibiotic treatment before semen analysis were considered as the patient group.
2009; Gallegos – Avila G. et al	Prospective cohort	2b	Men from couples who were attending the andrology infertility clinic with diagnosed genitourinary infection from Chlamydia trachomatis and Mycoplasma were included in the study. The age of men ranged from 25-51 years.
2009; A El feky	Case control	3b	Men with leycocytospermia, who were attending the out- patient infertility clinic in the department of Dermatology, Venereology and Andrology, Assiut University Hospital during June 2007 to May 2008, were included in the study. Their age ranged from 24 to 49 years old.
2010 Kokab A. et al	Prospective cohort	2b	Consecutive men who were attending the Avesina Research Institute in Tehran, Iran for diagnostic semen analysis were included in the study. All men were undergoing semen analysis as a part of a work-up for infertility investigations with their partner after failing to conceive after 1 year of unprotected intercourse. None of them reported any symptoms of genital tract infections.
2011; De Francesco M.A.	Retrospective cohort	2b	Men who were referred to the Microbiology Laboratory Group of Brescia’s main hospital for semen analysis, as a part of infertility work-up were included in the study. Patients had visited the clinic between 1 January 2004 and 31 December 2008.
2012; Rybar R. et al	Prospective cohort	2b	Men with no urogenital tract discomfort with a minimum sexual abstinence of 2 days were included in the study.
2013; Lee J.S.	Prospective study	2b	Male partners from infertile couples without female factor subfertility and without reproductive or hormonal abnormalities were included in the study.
2015; Huang C. et al	Prospective study	2b	Men of infertile couples who visited the Reproductive Center, the Reproductive and Genetic Hospital of CITIC, Xiangya, China, from January to December 2014 were included in the study. The men had failed to impregnate their wives after at least one year of unprotected intercourse.

**Figure 2:**
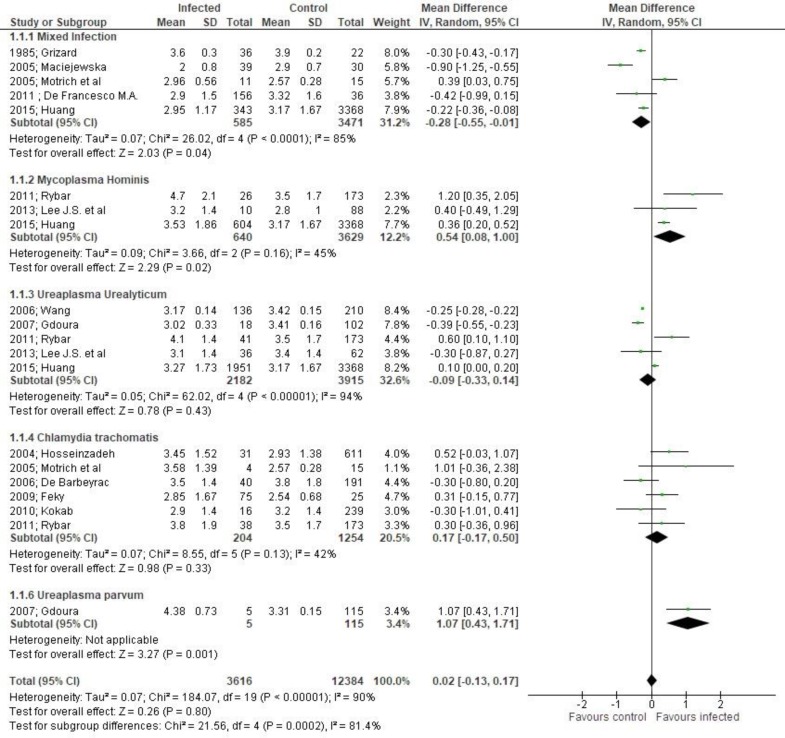
Mean differences of total sperm volume according to presence of bacteriospermia. The overall effect was not statistically significant (p = 0.80). (Vertical line = "no difference" point between two groups. Squares = mean differences; Diamonds = pooled mean differences for all studies. Horizontal lines = 95% CI).

**Figure 3:**
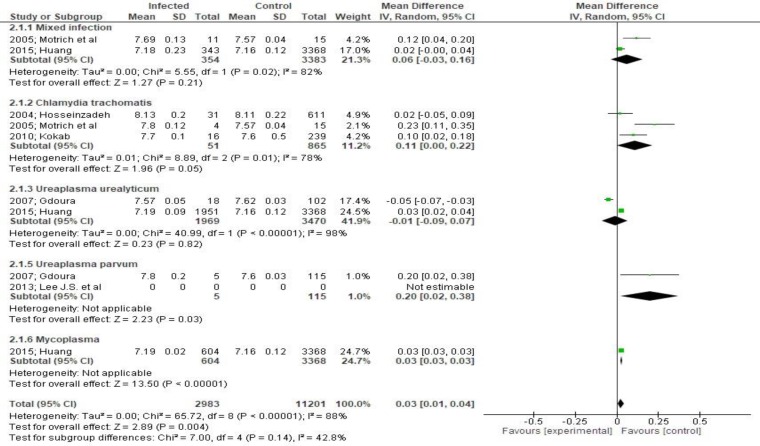
Mean differences in pH according to presence of bacteriospermia. The overall effect was statistically significant (p < 0.001). (Vertical line = "no difference" point between two groups. Squares = mean differences; Diamonds = pooled mean differences for all studies. Horizontal lines = 95% CI).

***Outcomes:*** Total sperm volume was not affected by the presence of bacteriospermia when all pathogens were analyzed together (MD 0.0295%CI -0.13,0.17, Figure 2). However, mixed infection, Mycoplama hominis and Ureaplasmaparvum may be associated with decreased volume (evidence from eight studies).A significant increase of pH was observed (MD 0.03 95%CI 0.01, 0.04, Figure 3).

**Figure 4:**
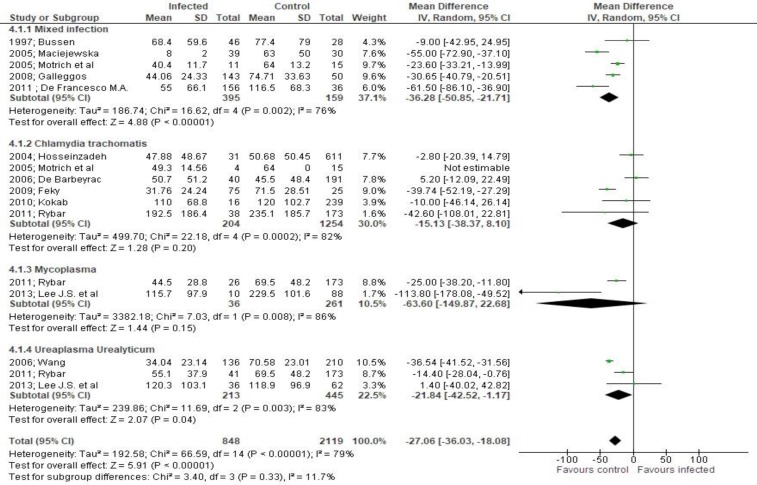
Mean differences of sperm concentration according to presence of bacteriospermia. The overall effect was statistically significant (p < 0.001). (Vertical line = "no difference" point between two groups. Squares = mean differences; Diamonds = pooled mean differences for all studies. Horizontal lines = 95% CI).

**Figure 5:**
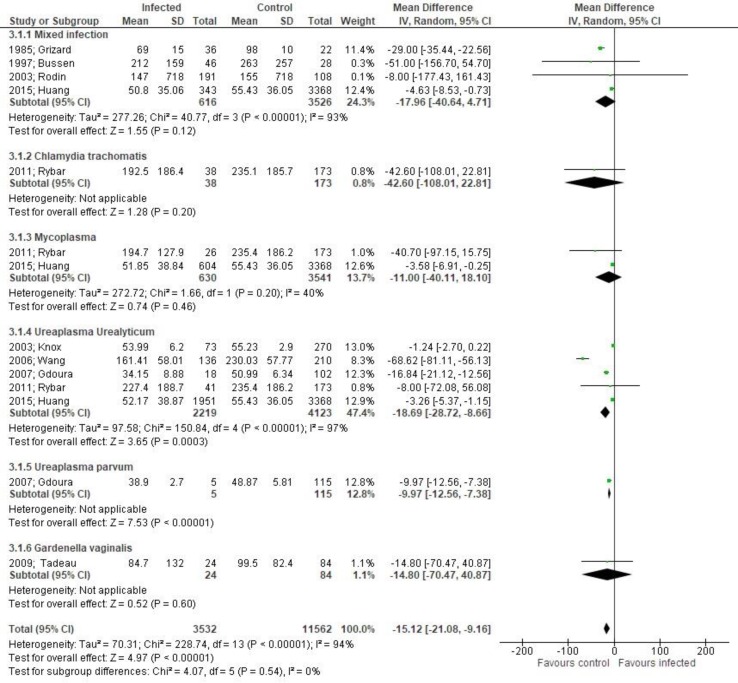
Mean differences of total sperm count according to presence of bacteriospermia. The overall effect was statistically significant (p < 0.001). (Vertical line = "no difference" point between two groups. Squares = mean differences; Diamonds = pooled mean differences for all studies. Horizontal lines = 95% CI).

**Figure 6:**
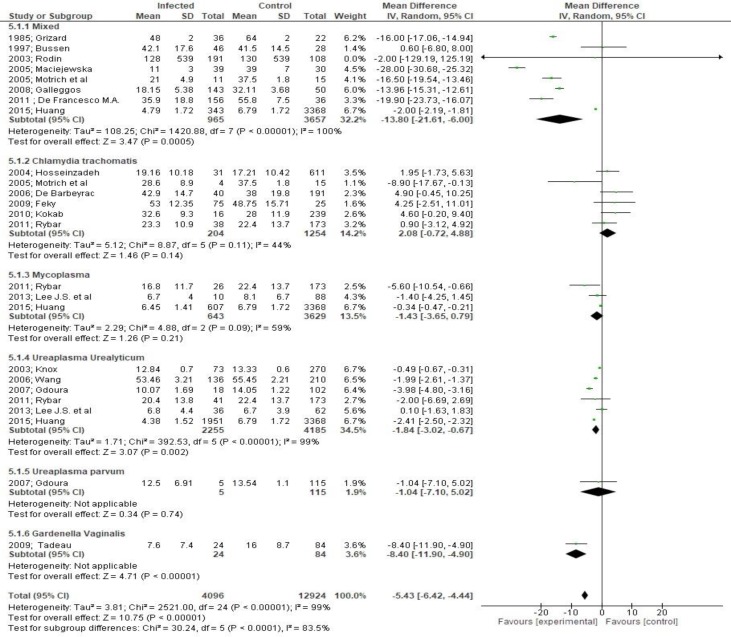
Mean differences in normal sperm morphology according to presence of bacteriospermia. The overall effect was statistically significant (p < 0.001). (Vertical line = "no difference" point between two groups. Squares = mean differences; Diamonds = pooled mean differences for all studies. Horizontal lines = 95% CI).

**Figure 7:**
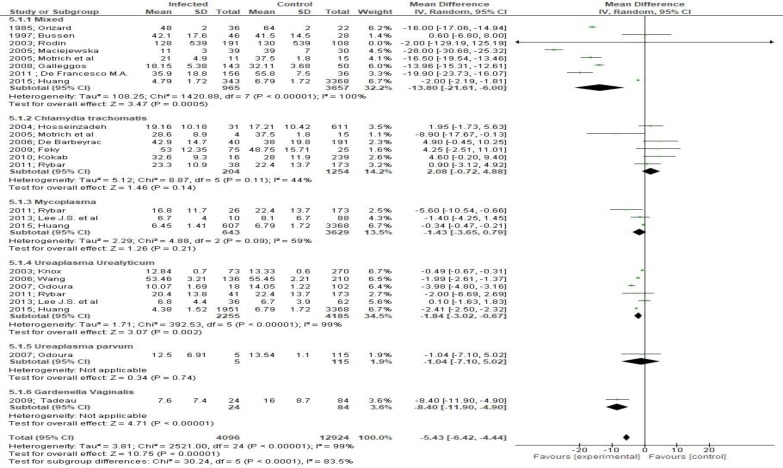
Mean differences in percentage of alive cells according to presence of bacteriospermia. The overall effect was statistically significant (p < 0.001). (Vertical line = "no difference" point between two groups. Squares = mean differences; Diamonds = pooled mean differences for all studies. Horizontal lines = 95% CI).

Both sperm concentration (MD -27.06, 95% CI -36.03, -18.08, Figure 4) and total sperm count (MD -15.12, 95% CI -21.08, -9.16, Figure 5) were significantly affected by bacteriospermia. Decreased rates of normal sperm morphology were also found (MD -5.43%, 95% CI -6.42, -4.44, Figure 6). The percentage of alive sperm was also affected by bacteriospermia (MD -4.39 %, 95% CI -8.25, -0.53, Figure 7).

**Figure 8:**
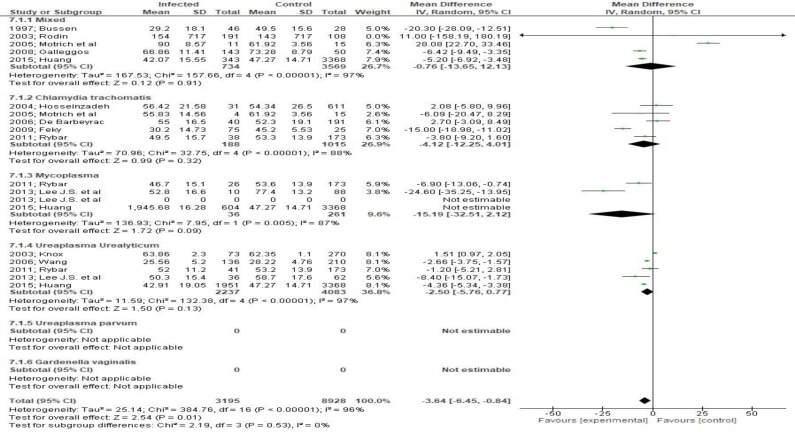
Mean differences in sperm motility according to presence of bacteriospermia. The overall effect was statistically significant (p < 0.01). (Vertical line = "no difference" point between two groups. Squares = mean differences; Diamonds = pooled mean differences for all studies. Horizontal lines = 95% CI).

**Figure 9:**
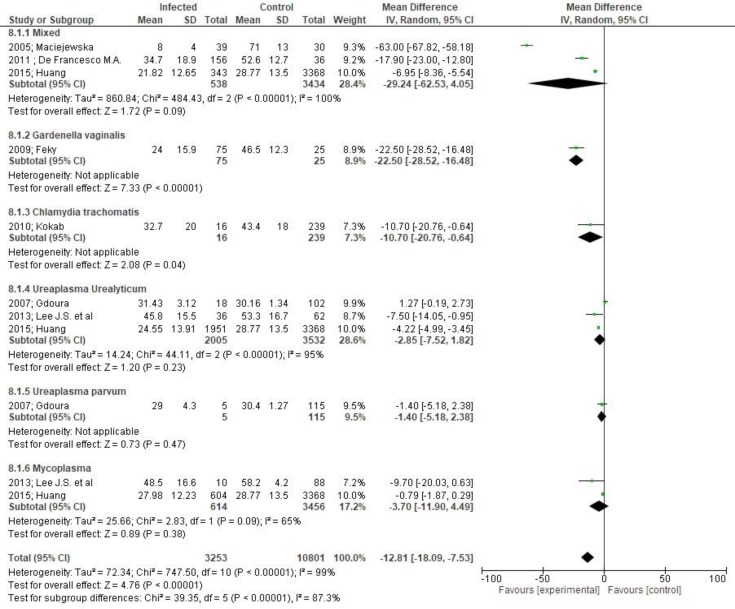
Mean differences in progressive cell motility according to presence of bacteriospermia. The overall effect was statistically significant (p<.001). (Vertical line = "no difference" point between two groups. Squares = mean differences; Diamonds = pooled mean differences for all studies. Horizontal lines = 95% CI).

The assessment of motility parameters revealed that total motility was quite affected by bacteriospermia (MD -3.64, 95 CI -6.45, - 0.84 Figure 8). Moreover, progressive motility was alsoaffected significantly (MD -12.81, 95% CI -18.09, -7.53, *p < 0.001 *Figure 9) an effect that was not, however, evident in patients with Ureaplasma infection. 

***Sensitivity analysis:*** The findings of the sensitivity analysis did not significantly affect the aforementioned results.

## Discussion

Both acute and chronic infections of the genitourinary tract are considered significant causative factors in male infertility (4). 

The effect of bacteriospermia in semen quality is not fully understood as the accurate pathophysiologic impact of the various bacteria in semen parameters remains vague (7). 

To date, to our knowledge, no previous meta-analyses in the field have been undertaken. In our meta-analysis we sought to gather all available evidence from the international literature to evaluate the influence of bacteriospermia in semen quality and male infertility. 

According, to our findings significantly decreased rates have been found in several parameters such as total sperm concentration, total sperm count, normal morphology and progressive motility. 

During the last decades, several studies have investigated the contribution of male factor and especially the role of bacteriospermia in couples’ infertility. Both symptomatic and asymptomatic bacteriospermia is associated with both acute and chronic inflammation of the genitourinary tract. Specifically, inflammatory mediators, such as cytokines and reactive oxygen species, restrain the normal function of Sertoli cells leading to restricted spermatogenesis and unsuccessful acrosome reaction. As a consequence, many couples due to various unsuccessful efforts of automatic pregnancy, are led to in vitro fertilization. However, because of reduced inducibility of acrosome reaction, in vitro fertilization efforts often fail (43). Thus, appropriate antibiotic treatment, according to Bieniek et al., is necessary to improve semen characteristics and reduce couple subfertility rates. 

***Implications for current clinical practice and future research:*** According to the findings of the present systematic review, bacteriospermia clearly affects several semen parameters. 

However, the impact of the various bacteria seems to differ. The clinical symptomatology does not necessarily correlate with the severity of these symptoms, as mild pathogens such as mycoplasma spp. may lead to significant alterations. Given these, clinicians should perform routine semen cultures when evaluating infertile couples and treat potential infections, despite the lack of substantial evidence for the effect of antibiotics on semen parameters. 

Taking in mind the gaps in current literature, we strongly believe that future studies are needed to determine clearly the accurate impact of symptomatic or asymptomatic bacteriospermia in semen characteristics and to evaluate the effect of the various bacteria. Furthermore, given the lack of clinical data in the field of antibiotic treatment and fertility outcomes, future randomized trials will help us to evaluate the impact of the various antibiotics and compare them to placebo. 

***Strengths and limitations of the study:*** Our study is based in a meticulous review of the current literature as we investigated thoroughly the majority of electronic databases and the grey literature. However, selection bias partially limits interpretation of our findings as the majority of included studies did not use data from the general population, but rather couples attending IVF centers; hence, it remains unclear whether the actual differences reflect the truth in the general population.

## Conclusion

Bacteriospermia seems to deteriorate semen parameters from normal values. However, current data are very limited as well as our understanding on the impact of antibiotic therapy on semen values. Future studies should focus on the impact of the various bacteria to corroborate our findings and enhance our knowledge in the pathophysiology and treatment of male infertility.
